# Genetic Diversification and Resistome of Coagulase-Negative Staphylococci from Nostrils of Healthy Dogs and Dog-Owners in La Rioja, Spain

**DOI:** 10.3390/pathogens13030229

**Published:** 2024-03-05

**Authors:** Idris Nasir Abdullahi, Carmen Lozano, Carmen González-Azcona, Myriam Zarazaga, Carmen Torres

**Affiliations:** 1 Area of Biochemistry and Molecular Biology, One-Health-UR Research Group, University of La Rioja, 26006 Logroño, Spain; idris-nasir.abdullahi@unirioja.es (I.N.A.); carmen.lozano@unirioja.es (C.L.); carmen.gonzalezaz@unirioja.es (C.G.-A.); myriam.zarazaga@unirioja.es (M.Z.); 2 Department of Medical Laboratory Science, Faculty of Allied Health Sciences, College of Medical Sciences, Ahmadu Bello University, Zaria 810107, Nigeria

**Keywords:** CoNS, pet staphylococci, AMR, linezolid resistance, zoonosis

## Abstract

Coagulase-negative staphylococci (CoNS) species in healthy dogs and their owners could be transferred between these hosts and carry diverse antimicrobial resistance (AMR) genes of public health concern. This study determined the frequency, diversity, and AMR genes of nasal CoNS from healthy dogs and in-contact people as well as the rate of intra-household (between healthy dogs and dog-owners) transmission of CoNS. Nasal samples were collected and processed from 34 dogs and 41 humans from 27 households, and CoNS identification was done by MALDI-TOF-MS. The AMR determinants and genetic lineages were determined by PCR/sequencing. A total of 216 CoNS isolates were initially obtained and identified, and the AMR phenotypes were determined. From these, 130 non-repetitive CoNS were selected (one isolate of each species per sample or more than one if they presented different AMR phenotypes) and further characterized. The predominant species from dog carriers were *S. epidermidis* (26.5%), *S. hominis* (8.8%), and *S. cohnii* (8.8%), whereas in the human carriers, the predominant ones were *S. epidermidis* (80.4%), *S. lugdunensis* (9.8%), and *S. hominis* (9.8%). Intra-host species diversity (>one CoNS species) was detected in 37.5% of dogs and 21.6% of dog-owners. Conversely, 50% of dogs and 70.3% of dog-owners had intra-species AMR diversity (2–4 AMR-CoNS profiles). About 20% were susceptible to all antimicrobial agents tested, 31.5% displayed a multidrug resistance phenotype, and 17.4% were *mecA*-positive, located in SCC*mec* type V (24.2%), III (18.1%), IVc (12.1%), and II (6.1%). The other *mec-A* positive CoNS isolates (39.5%) had non-typeable SCC*mec*. The highest AMR rates were found against erythromycin (32.3%/*mph*(C), *msr*(A)) and mupirocin (20.8%/*mupA*), but the resistance rates for other antimicrobial agents were <10% each. Remarkably, one linezolid-resistant *S. epidermidis*-ST35 isolate was identified and mediated by four amino acid substitutions in L3 and one in L4 ribosomal proteins. Dogs and dog-owners as carriers of *S. epidermidis* with similar AMR patterns and genetic lineages (ST59, ST61, ST166 and ST278) were detected in four households (14.8%). Diverse CoNS carriage and moderate level of AMR were obtained from this study. The detection of CoNS carrying diverse SCC*mec* elements and intra-species AMR diversity highlights the roles of dog ownership in the potential transmission of antimicrobial-resistant CoNS in either direction.

## 1. Introduction

Recently, coagulase-negative staphylococci (CoNS) species have been attracting public health interest as they have been implicated in healthcare-associated infections, with substantial impacts on human and animal health [1,2]. However, they are rarely reported in community-associated infections [1,2]. *Staphylococcus epidermidis* is the most common CoNS responsible for various clinical conditions ranging from prosthetic device-associated infections and neonatal sepsis due to its high strain-level heterogeneity [3,4,5]. *S. lugdunensis* is another CoNS species that causes a wide range of infections like those of *Staphylococcus aureus* [6]. Particularly, it is more virulent than other CoNS and has been confirmed to be responsible for life-threatening infective endocarditis [7]. Moreover, *S. lugdunensis* is a good bacteriocin producer (lugdunin) against many Gram-positive cocci such as *S. aureus* [8,9]. Although sparsely reported, some CoNS species (such as methicillin-resistant *S. haemolyticus* and *S. epidermidis*) have been identified as etiological agents for infections in dogs [10,11,12], but their zoonotic potential and relevance in canine health need to be determined.

The ability of CoNS species to notoriously acquire antimicrobial resistance (AMR) genes and produce biofilms on inanimate surfaces makes them very difficult to treat [13,14]. Moreover, the methicillin-resistant CoNS (MRCoNS) isolates are often associated with additional AMR genes which may facilitate the risk of gene transfer between MRCoNS with other *Staphylococcus* species with higher pathogenic properties through mobile genetic elements [15]. Also, multidrug resistant-CoNS could significantly limit the availability of treatment options for staphylococcal infections of humans and animals, especially if they carry critical AMR genes such as those that mediate linezolid resistance [16,17].

Coagulase-positive staphylococci (CoPS) can be exchanged in dog-owning households as demonstrated by our recent study [18]. However, few data are available on the incidence and diversity of CoNS in healthy dogs and their owners at the household level and the potential cases of interhost transmission. Among the few available studies is a previous study conducted by our research group, in which samples were collected over a decade ago to determine the potential influence of dog ownership on the concomitant nasal carriage of more than one CoNS species and transmission between dogs and their owners [19]. This present study fundamentally evaluated the current situation in the same area (La Rioja, Spain), specifically to determine the ecology and resistome diversity of CoNS obtained from the nasal cavities of clinically healthy dogs and in-contact people, and to classify the Staphylococcal Cassette Chromosome *mec* (SCC*mec*) mobile elements of methicillin-resistant-CoNS and determine the intra-household (between healthy dogs and dog-owners) transmission of *S. epidermidis*.

## 2. Materials and Methods

### 2.1. Study Participants and Bacterial Recovery and Identification

The description of the enrolled individuals (dogs and dog-owners) and how the nasal samples were obtained is provided in the previous study in which the prevalence of CoPS was addressed [18]. However, this present study was focused on the isolation and identification of CoNS from 34 dogs and 41 dog-owners in 27 unrelated households of the La Rioja region in northern Spain. All isolates with morphological characteristics of staphylococci were identified by MALDI-TOF-MS as previously described [20]. The protocols and methodology used in this study have been reviewed and approved by the ethical research committee of the University of La Rioja (Spain). In this regard, the collection, processing, and analyses of human and animal samples were carried out following all applicable international, national, and/or institutional guidelines for human experiments (as described in the revised Helsinki declaration) and for ethical use of animals (directive 2010/63/EU, Spanish laws 9/2003 and 32/2007, and RD 178/2004 and RD 1201/2005).

### 2.2. Antimicrobial Susceptibility Tests

All identified CoNS isolates were tested for their antimicrobial susceptibility to 13 agents by the agar disk diffusion method following the recommendations and breakpoints provided by the European Committee on Antimicrobial Susceptibility Testing [21]. The antimicrobial agents tested were as follows (µg/disk): penicillin (10), cefoxitin (30), gentamicin (10), tobramycin (10), erythromycin (ERY, 15), clindamycin (CLI, 2), chloramphenicol (30), linezolid (10), mupirocin (200), trimethoprim-sulfamethoxazole (1.25 + 23.75), tetracycline (30), and ciprofloxacin (5). Also, the minimum inhibition concentration (MIC) of linezolid in the linezolid-resistant CoNS (initially detected by disc diffusion) was determined by Etest (BioMérieux Linezolid Etest^®^, Marcy l’Étoile, France) and results were interpreted as per the recommendation of the test manufacturer and EUCAST 2022. Inducible resistance to clindamycin was tested by the ‘D test*’*. In this regard, the ERY and CLI disks were placed together (12 mm apart). Isolates that were resistant to ERY and susceptible to CLI but showed a D-shaped distorted zone of inhibition around CLI under the influence of ERY were considered ERY-CLI inducible resistance.

Multidrug resistance (MDR) in the CoNS isolates was defined by the presence of resistance to ≥3 classes of antimicrobial agents used in human and veterinary medicine [22,23]. All non-repetitive CoNS isolates (isolates of different samples or those of the same sample but with different species or different AMR phenotypes) were further characterized.

### 2.3. Characterization of Antimicrobial Resistance Determinants

The mechanism of AMR in all non-repetitive CoNS isolates was tested by PCR and confirmed by sequencing (when required). The following genes were selected based on the type and class of antimicrobial agents: beta-lactams (*blaZ*, *mecA*), aminoglycosides (*aac6′-aph2″* and *ant4′*), tetracycline (*tet*(K), *tet*(L), *tet*(M)), erythromycin and clindamycin (*erm*(A), *erm*(B), *erm*(C), *erm*(T), *lnu*(A), *lnu*(B), *vga*(A), *msr*(A), *mph*(C)), chloramphenicol (*fexA*, *fexB*, *catA*, *cat_pC194_*, *cat_pC221_*, and *cat_pC223_*), trimethoprim-sulfamethoxazole (*dfrA*, *dfrD*, *dfrG*, *dfrK*), mupirocin (*mupA*), and linezolid (*cfr*, *cfrD*, *optrA*, and *poxtA*). Primers and conditions of PCRs for the AMR genes tested are included in Supplementary Table S1. Moreover, mutations in 23S rRNA were investigated by PCR/sequencing. Also, amino acid substitutions on the 50S ribosomal proteins L3 (*rplC*), L4 (*rplD*), and L22 (*rplV*) were screened on the linezolid-resistant CoNS by PCR/sequencing (Supplementary Table S1). The obtained sequences were compared with those of *S. epidermidis* ATCC12228 (GenBank accession number CP022247) using the EMBOSS Needle software (https://www.ebi.ac.uk/jdispatcher/psa/emboss_needle, accessed on 12 February 2023) for nucleotide or amino acid (BLOSUM 62 cost matrix) alignments.

### 2.4. Molecular Typing of S. epidermidis and MRCoNS Isolates

The sequence types of all *S. epidermidis* isolates with similar antimicrobial susceptibility profiles from hosts (dogs and dog-owners) of the same households were determined by MultiLocus Sequence Typing (MLST). The seven housekeeping genes of *S. epidermidis (acrC*, *aroE*, *gtr*, *pyrR*, *mutS*, *tpi*, and *yqiL*) were amplified, and the sequence type (ST) was assigned according to the MLST database (https://pubmlst.org/, accessed on 23 April 2023). Moreover, SCC*mec* typing of all non-repetitive MRCoNS was performed by multiplex PCRs as previously described [24]. Primers and conditions of PCRs for the AMR genes tested are included in Supplementary Table S1. Positive controls (confirmed by sequencing) from the collection of the University of La Rioja were included in all PCRs in this study.

## 3. Results

### 3.1. Frequencies and Species Diversity of Coagulase-Negative Staphylococci in Healthy Dogs and Dog-Owners

A total of 216 CoNS were recovered from the 34 dogs and 41 dog-owners of the 27 households tested in this study (up to six isolates per positive sample), and the distribution of species is indicated in Table 1. After species identification and AMR phenotype determination, a collection of 130 non-repetitive CoNS isolates was obtained and genetically characterized and considered in this study. These 130 CoNS isolates corresponded to one isolate of each species per sample or more than one if they presented different AMR phenotypes (Table 1).

A total of 32 non-repetitive CoNS isolates were identified from 16 of the 34 dogs (of nine species: *S. epidermidis*, *S. hominis*, *S. cohnii*, *S. pasteuri*, *S. warneri*, *S. xylosus*, *S. haemolyticus*, *S. simulans*, and *S. muscae*) (Table 1). In addition, 98 non-repetitive isolates were identified from 37 of 41 dog-owners (of six species: *S. epidermidis*, *S. lugdunensis*, *S. hominis*, *S. pasteuri*, *S. warneri*, and *S. xylosus*) (Table 1). About 41.7% and 90.2% of dogs and dog-owners carried at least one CoNS species, respectively (Table 1). The predominant species from dog carriers were *S. epidermidis* (26.5%), *S. hominis* (8.8%), and *S. cohnii* (8.8%); whereas in the human carriers, the predominant ones were *S. epidermidis* (80.4%), *S. lugdunensis* (9.8%), and *S. hominis* (9.8%) (Table 1).

### 3.2. Antimicrobial Resistance Phenotypes and Genotypes of Non-Repetitive CoNS Isolates

**Figure 1:**
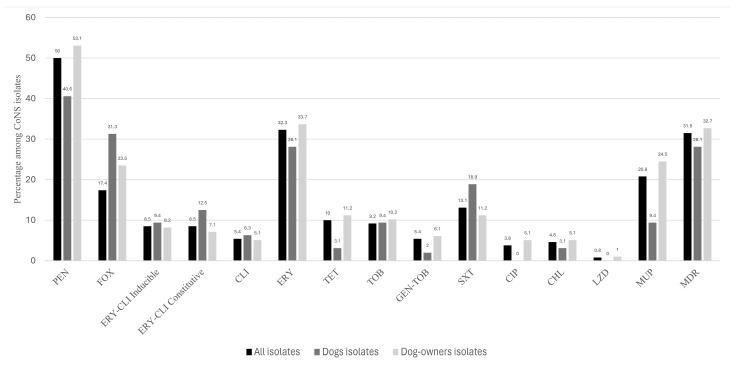
Frequency of antimicrobial resistance among non-repetitive coagulase-negative staphylococci isolates from nasal cavities of healthy dogs and dog-owners. CHL: chloramphenicol; CLI: clindamycin; CIP: ciprofloxacin; ERY: erythromycin; FOX: cefoxitin; GEN: gentamicin; LZD: linezolid; MUP: mupirocin; MDR: multi-drug resistance phenotype; PEN: penicillin; SXT: sulfamethoxazole/trimethoprim; TET: tetracycline, TOB: tobramycin.

**Figure 2:**
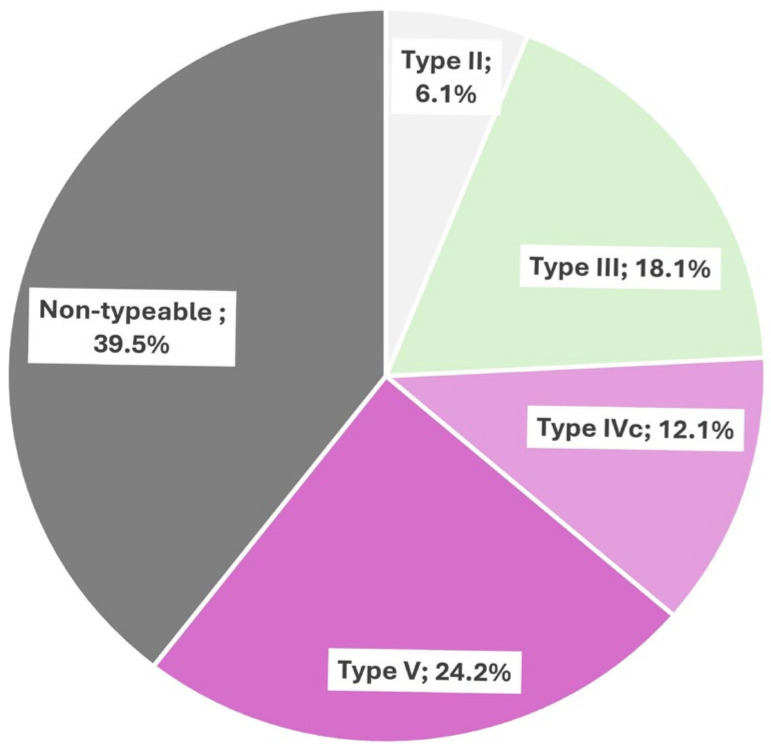
Frequency of the SCC*mec* mobile elements identified among the 33 non-repetitive methicillin-resistant coagulase-negative staphylococci isolates in healthy dogs and dog-owners.

Of the 32 and 98 non-repetitive CoNS from dogs and dog-owners, 21.9% and 19.4% were susceptible to all antimicrobial agents tested, respectively. Also, 34.4% and 26.5% of the isolates from dogs and dog-owners, respectively, were resistant to only one antimicrobial agent tested (Table 1). However, 28.1% and 32.7% of the isolates from dogs and dog-owners presented an MDR phenotype. Collectively, 20% were susceptible to all antimicrobial agents tested, while 31.5% carried multidrug resistance (MDR) phenotypes (Table 1). The rates of resistance detected in the collection of 130 CoNS isolates were as follows [percentage of resistance/detected genes, mutation, or amino acid substitution]: penicillin [50/*blaZ*], cefoxitin (17.4/*mecA*), erythromycin–clindamycin-inducible [8.5/*erm*(A)], erythromycin–clindamycin-constitutive [8.5/*erm*(C), *erm*(T)], erythromycin [32.3/*mph*(C), *msr*(A)], clindamycin [5.4/*vga*(A), *lsaB*], tobramycin [9.2/*ant4′*], gentamicin–tobramycin [5.4/*aac6′-aph2″*), tetracycline [10/*tet*(K), *tet*(M)], sulfamethoxazole–trimethoprim [13.1/*dfrA*, *dfrG*], chloramphenicol [4.6/*catPC221*], mupirocin [20.8/*mupA*], linezolid [0.8/four amino acid substitutions in L3 and one in L4 ribosomal proteins] and ciprofloxacin [3.8] (Table 2 and Table 3 and Figure 1). The *erm(T)* gene was detected only in *S. epidermidis* and *S. hominis* isolates of dog-owners (Table 2). Moreover, one of the four *S. lugdunensis* isolates identified in four dog-owners carried an MDR phenotype (PEN-ERY-MUP/*blaZ, erm*(A)*, mupA*), two others were only resistant to PEN (*blaZ*), and the remaining one was susceptible to all antimicrobial agents tested (Table 2 and Table 3). The linezolid-resistant *S. epidermidis* isolate had an MIC for this antimicrobial agent of 16 μg/mL and resistance was mediated by amino acid substitutions on 50S ribosomal protein L3 (Ile188Val, Gly218Val, Asp219Ile, Leu220Asp) and L4 (Asn158Ser) (Supplementary Figure S1a,b). Among the 33 MRCoNS, all were *mecA*-positive, but only 20 were associated with a specific SCC*mec* type. In this regard, the SCC*mec* type V element was the predominant (24.2%), followed by SCC*mec* type III (18.1%), SCC*mec* type IVc (12.1%), and SCC*mec* type II (6.1%). The other *mec-A* positive CoNS isolates (39.5%) had non-typeable SCC*mec* (Figure 2).

### 3.3. Intra-Host Species Diversity of Coagulase-Negative Staphylococci in Healthy Dogs and Dog-Owners

**Figure 3:**
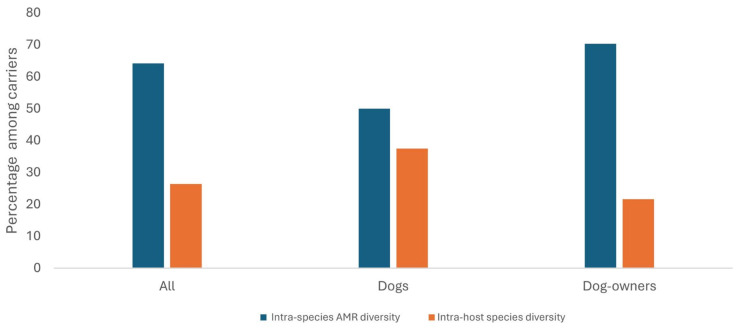
Frequency of intra-host species and intra-species AMR diversity among non-repetitive coagulase-negative staphylococci from healthy dogs and dog-owners. Note: The number of hosts with nasal carriage of more than one CoNS species were 16 dogs and 37 dog-owners.

Intra-host species diversity (more than one CoNS species in a sample) was detected in 37.5% of dogs and 21.6% of dog-owners (Figure 3 and Table 4). In one of the dog-owners (number 26 in household 11) carrying diverse isolates, three heterogeneous *S. epidermidis* and two *S. hominis* isolates carrying different resistomes were identified (Table 4). Also, in dog-owner 66 of household 25, three heterogeneous *S. epidermidis* and one *S. lugdunensis* isolates carrying different antimicrobial resistance genes were identified (Table 4). In dog 18 of household 8, five different isolates of *S. epidermidis*, *S. haemolyticus*, and *S. warneri* carrying both methicillin-resistant and methicillin-susceptible traits were identified (Table 4). Conversely, 50% of dogs and 70.3% of dog-owners had intra-species AMR diversity (2–4 varied AMR profiles) (Figure 3).

### 3.4. Intra-Household Carriers of Similar S. epidermidis Isolates and Their Genetic Lineages

Dogs and dog-owners that were carriers of *S. epidermidis* with similar AMR patterns and genetic lineages were detected in four households (14.8%). In two of these households, the *S. epidermidis* were susceptible to all antimicrobial agents tested while the other *S. epidermidis* isolates from the three households were resistant to only clindamycin (Table 5). The genetic lineage of *S. epidermidis* in the carriers in households 3, 6, 11, 13, and 19 were ST59, ST61, ST166, and ST278.

## 4. Discussion

There are few available data on the nasal carriage of CoNS, especially in healthy pets and their owners. Aside from the previous study by Gómez-Sanz et al. [19], we are not aware of any similar comprehensive study on the nasal staphylococci microbiota and intra-species heterogeneity of AMR in these hosts. In the present study, several CoNS species were isolated from the two hosts, but they were more diverse in healthy dogs than in their owners. This is similar to the report from a study in Trinidad where significantly more diverse species of CoNS were identified from healthy dogs than from the owners [25]. However, the CoNS species widely differ between our study and that of Suepaul et al. [25], perhaps due to the type of dog breed or the geographical/hygienic status of the hosts. Most of the CoNS isolated in the present study belonged to the species *S. epidermidis* in both dogs and their owners. *S. epidermidis* is the most reported among CoNS in humans [26] and has often been detected in healthy pets [27]. Being the major nasal commensal in humans, the predominance of *S. epidermidis* is not surprising in the dog-owners. Although at lower rates, it is also a predominant species in healthy dogs [19,27] perhaps due to the influence of human–pet direct or indirect contact in their household. It is important to note that *S. epidermidis* is largely not a normal microbiota in dogs.

Aside from *S. epidermidis*, *S. hominis* was identified at a moderate rate in both hosts. Reports on the carriage of *S. hominis* in healthy dogs and humans are scarce; the same is true for other species identified at low rates (*S. haemolyticus*, *S. pastueri*, and *S. warneri*). A recent similar study reported the same trend, but in nasal skin samples of healthy dogs [28]. Remarkably, *S. lugdunensis* was identified only in four dog-owners but not in dogs. This is a very relevant CoNS species in humans causing diverse clinical infections [9]. None of the dogs was colonized by this species; however, colonized dog-owners could place their dogs at risk of anthroponotic infections, as has previously been implicated in canine infection [29].

Concerning the AMR profile of the CoNS in this study, *mecA*-positive CoNS (carried by diverse SCC*mec* elements) was found in 23.2% of the isolates of healthy dog-owners and is slightly higher than those detected in studies among clinically healthy people (less than 20%) [25,30,31]. However, a slightly higher rate of 27.9% was reported from dog-owners in Spain [19] and much higher rates (>50%) have previously been reported in studies on Asian children and a remote population in French Guiana [32,33]. Conversely, the carriage rate of MR-CoNS obtained in this study was similar to a multinational study on hospital workers (21.4%) [34]. Put together, these data indicate the variation in nasal carriage of MR-CoNS based on occupation, age, geographical region, and contact with animals. However, it is important to note that studies on MRCoNS from dog-owners are particularly sparse. Most of the MRCoNS from our study were of the species *S. epidermidis,* others include *S. hominis* in both dogs and owners, but *S. cohnii* only in dogs. The MR-*S. cohnii* and MR-*S. hominis* are rarely isolated from healthy dogs. If we consider the comparably similar types of SCC*mec* elements in some of these MRCoNS isolates, one could suggest the potential transfer of the *mecA* gene to these non-*epidermidis*-MRCoNS isolates.

Half of the CoNS from dogs and their owners were penicillin-resistant, mediated by the *blaZ* gene that produces a beta-lactamase. A similar rate was reported by Seupaul et al [25]. This is not surprising, as penicillin is one of the first-line antimicrobial agents, and its frequent use may contribute to selection pressures in the *Staphylococcus* spp. Moreover, various forms of macrolide–lincosamide–streptogramin (MLS) resistance phenotypes/genotypes such as the erythromycin-resistant-clindamycin-susceptible (by *msr*(A)*, mph*(C)), erythromycin–clindamycin-constitutive (by *erm*(A) and *erm*(T)), erythromycin–clindamycin-inducible (by *erm*(C)), and erythromycin-susceptible–clindamycin-resistant (by *vga*(A)*, lsa*(B)) were exhibited by over 50% of the CoNS. These classes of antimicrobial agents are relevant treatment options in most clinical staphylococcal infections [35,36]. Hence, the AMR to this category of drugs is of serious concern. Of note is the detection of the *erm*(T) gene in *S. epidermidis* and *S. hominis*. This is an unusual mechanism of erythromycin–clindamycin-constitutive resistance that appears to be evolving in humans and animals.

Resistance to aminoglycosides and chloramphenicol has been reported at very low rates. Aminoglycosides are used extensively in clinical settings [27,35,36], and chloramphenicol is rarely in use in humans or pets. Perhaps this is why the *fexA* and *fexB* genes were not detected among the chloramphenicol-resistant isolates; rather, *cat*_pC221_ was detected, which appears to be one of the common mechanisms of resistance in non-*aureus* staphylococci. Most important is the detection of linezolid-resistant *S. epidermidis* isolate mediated by multiple amino acid substitutions on L3 and one on L4 ribosomal proteins. This is the first report on this mechanism of linezolid resistance in *S. epidiermidis*-ST35 in the literature. These mechanisms of resistance are not transferable to other species or bacteria but confirm the silent and slow emergence of high-level resistance in CoNS in dog-owners.

Tetracycline resistance was generally detected at a moderate level but relatively more in dog-owners than in dogs. Most studies on mupirocin resistance focused on *S. aureus* isolates and very few data are available on the CoNS species [19], especially from healthy pets and their owners. In this study, the mupirocin resistance rate was high among the CoNS from both hosts, and this is a cause for concern, as mupirocin has long been used in the nasal decolonization of *S. aureus* [37].

MDR was high in both dogs and dog-owners, but slightly less in dogs than in their owners. MDR from healthy pets and their owners may limit the available treatment options for staphylococcal infections in humans and animal medicine [38]. Another important phenomenon to remark on is the high intra-host species and intra-species AMR diversity. Being heterogeneous, more *S. epidermidis* exhibited intra-species diversity with varied AMR genes. This may pose a difficulty in eradicating *S. epidermidis*, especially in prosthetic joint infections [5,39]. The potential transmission of *S. epidermidis* between owners and their pets was detected in five households where the dogs and dog-owners were colonized by similar CoNS species with the same genetic lineages. Similar findings were previously reported in Spain but on MR-CoNS [19]. Put together, this highlights the transmission of *Staphylococcus* species other than *S. aureus* and *S. pseudintermedius* in dog-owning households.

## 5. Conclusions

Diverse CoNS carriage and moderate-level AMR were obtained from the hosts. The detection of MRCoNS carrying SCC*mec* elements, intra-host species diversity and linezolid resistant *S. epidermidis* highlight the need for the extension of AMR surveillance systems to CoNS and as well as dog-owning households. Also, the detection of nasal *S. epidermidis* of the same lineages and indistinguishable AMR genotypic in participants from the same household are good indicators that strains are exchanged between humans and their dogs (potential anthroponosis). However, the ultimate proof of exchange events would be the comparison of the whole genome sequences of the respective isolates.

## Figures and Tables

**Table 1:** Frequencies of coagulase-negative staphylococci from healthy dogs and dog-owners and characteristics of non-repetitive isolates ^a^.

CoNS Species	Total Isolates Recovered from Dogs and Dog-Owners	Non-Repetitive Isolates		No. of Carriers (%)		Non-Repetitive Isolates with the Following Characteristics						
						Susceptible to All Antimicrobial Agents (%)		Resistance to Only One Antimicrobial Agent (%)		MDR Phenotype (%)		
		Dogs	Dog-Owners	Dogs	Dog-Owners	Dogs	Dog-Owners	Dogs	Dog-Owners	Dogs	Dog-Owners	Total
*S. epidermidis*	167	12	82	9 (26.5)	33 (80.4)	3 (25)	14 (17.1)	4 (33.3)	22 (26.8)	4 (33.3)	29 (35.6)	33 (35.1)
*S. hominis*	13	3	5	3 (8.8)	4 (9.8)	1 (33.3)	1 (20)	1 (33.3)	1 (20)	2 (66.7)	2 (40)	4 (50)
*S. cohnii*	4	3	0	3 (8.8)	0	0	0	0	0	2 (66.7)	0	2 (66.7)
*S. lugdunensis*	5	0	4	0	4 (9.8)	0	1 (25)	0	1 (25)	0	1 (25)	1 (25)
*S. pasteuri*	7	1	4	1 (2.9)	3 (7.3)	1 (100)	2 (50)	0	1 (25)	0	0	0
*S. warneri*	9	5	2	2 (5.7)	2 (4.9)	0	1 (50)	2 (40)	0	1 (20)	0	1 (14.3)
*S. xylosus*	4	2	1	1 (2.9)	1 (2.4)	0	0	1 (50)	1 (100)	0	0	0
*S. haemolyticus*	3	3	0	2 (5.7)	0	1 (33.3)	0	1 (33.3)	0	0	0	0
*S. simulans*	3	2	0	2 (5.7)	0	1 (50)	0	1 (50)	0	0	0	0
*S. muscae*	1	1	0	1 (2.9)	0	0	0	1 (100)	0	0	0	0
Total (%)	216	32	98	16 (47.1)	37 (90.2)	7 (21.9)	19 (19.4)	11 (34.4)	26 (26.5)	9 (28.1)	32 (32.7)	41 (31.5)

^a^ Non-repetitive isolates are those of different individuals or of different species or different AMR phenotypes.

**Table 2:** Antimicrobial resistance phenotypes and genotypes in non-repetitive coagulase-negative staphylococci identified in healthy dog-owners.

Species with Antimicrobial Resistance in Dog-Owners	Antimicrobial Resistance among Isolates from Dog-Owners	
	Phenotype (Number of Isolates)	Genes or Amino Acid Substitutions Detected (Number of Isolates)
*S. epidermidis*, *S. pasteuri*, *xylosus*	PEN (49)	*blaZ* (46)
*S. lugdunensis*	PEN (3)	*blaZ* (3)
*S. epidermidis*, *S. hominis*	FOX (23)	*mecA* (23)
*S. epidermidis*, *S. hominis*	ERY-CLI constitutive (5)	*erm*(A) (5)
*S. epidermidis*, *S. hominis*	ERY-CLI constitutive (2)	*erm*(T) (2)
*S. epidermidis*	ERY-CLI inducible (8)	*erm*(C) (8)
*S. epidermidis*, *S. pasteuri*, *S. hominis*	ERY (33)	*msr*(A) (33)*, mph*(C) (6)
*S. lugdunensis*	ERY (1)	*msr*(A) (1)
*S. epidermidis*	CLI (5)	*vga*(A) (5), *lsa*(B) (1)
*S. epidermidis*	GEN-TOB (2)	*aac6′-aph2″* (2)
*S. epidermidis*	TOB (9)	*ant4′* (9)
*S. epidermidis, S. hominis*	GEN (4)	*aac6′-aph2″* (4)
*S. epidermidis*	CIP (5)	NT
*S. epidermidis*, *S. hominis*	TET (11)	*tet*(K) (11), *tet*(M) (1)
*S. epidermidis*	SXT (11)	*dfrA* (10), *dfrG* (5)
*S. epidermidis*	CHL (5)	*cat*_pC221_ (5)
*S. epidermidis*-ST35	LZD (1)-MIC 16 µg/mL	Amino acid changes in L3 (I188V, G218V, N219I, L220D) and L4 (N158S)
*S. epidermidis*, *S. hominis*, *S. warneri*	MUP (23)	*mupA* (23)
*S. lugdunensis*	MUP (1)	*mupA* (1)

CHL: chloramphenicol; CLI: clindamycin; CIP: ciprofloxacin; ERY: erythromycin; FOX: cefoxitin; GEN: gentamicin; LZD: linezolid; MUP: mupirocin; PEN: penicillin; SXT: sulfamethoxazole/trimethoprim; TET: tetracycline, TOB: tobramycin. NT: Not tested.

**Table 3:** Antimicrobial resistance phenotypes and genotypes in non-repetitive coagulase-negative staphylococci identified from healthy dogs.

Species with Antimicrobial Resistance in Dogs	Antimicrobial Resistance among Isolates from Dog	
	Phenotype (Number of Isolates)	Genes or Amino Acid Substitutions Detected (Number of Isolates)
*S. epidermidis*, *S. cohnii*, *S. simulans, xylosus*	PEN (13)	*blaZ* (13)
*S. epidermidis*, *S. cohnii*, *S. hominis*	FOX (10)	*mecA* (10)
*S. epidermidis*, *S. cohnii*	ERY-CLI constitutive (4)	*erm*(A) (4)
*S. epidermidis*, *S. hominis*	ERY-CLI inducible (3)	*erm*(C) (3)
*S. epidermidis*, *S. warneri*, *S. hominis*	ERY (9)	*msr*(A) (9)
*S. epidermidis*	CLI (2)	*vga*(A)(2)
*S. haemolyticus*	GEN-TOB (1)	*aac6′-aph2″* (1)
*S. hominis*, *S. xylosus*	TOB (3)	*ant4′* (3)
*S. haemolyticus*	TET (1)	*tet*(K) (1)
*S. epidermidis*, *S. warneri*, *S. hominis*	SXT (6)	*dfrA* (6)
*S. warneri*	CHL (1)	*cat*_pC221_ (1)
*S. epidermidis*	MUP (3)	*mupA* (3)

CHL: chloramphenicol; CLI: clindamycin; ERY: erythromycin; FOX: cefoxitin; GEN: gentamicin; LZD: linezolid; MUP: mupirocin; PEN: penicillin; SXT: sulfamethoxazole/trimethoprim; TET: tetracycline, TOB: tobramycin.

**Table 4:** Intra-host species and intra-species AMR diversity in coagulase-negative staphylococci from healthy dogs and dog-owners.

Host	Host ID/Household	Species	AMR Phenotype	AMR Genes Detected
Human	22/10	*S. pasteuri* *S. pasteuri* *S. epidermidis* *S. epidermidis*	Susceptible PEN-ERY PEN-TOB MUP	NT *blaZ*, *msr*(A) *blaZ*, *ant4′* *mupA*
	26/11	*S. epidermidis* *S. epidermidis* *S. epidermidis* *S. hominis* *S. hominis*	PEN PEN-ERY-CLI^ind^ PEN-ERY-CLI-MUP-LZD PEN-FOX-ERY-CLI-TET-MUP PEN-ERY-GEN-MUP	*blaZ* *blaZ*, *erm*(C) *blaZ*, *mecA*, *msr*(A), *mph*(C), *mupA* *blaZ*, *mecA*, *erm*(A), *tet*(K), *mupA* *blaZ*, *mecA*, *msr*(A), *aac6′-aph2″*, *mupA*
	27/11	*S. epidermidis* *S. lugdunensis* *S. hominis*	PEN-ERY-CLI-MUP PEN PEN	*blaZ*, *erm*(C), *mupA* *blaZ* *blaZ*
	35/14	*S. pasteuri* *S. epidermidis*	ERY CLI-FOX	*msr*(A) *lnuA*, *mecA*
	64/24	*S. xylosus* *S. epidermidis*	PEN Susceptible	*blaZ* NT
	66/25	*S. epidermidis* *S. epidermidis* *S. epidermidis* *S. lugdunensis*	FOX-TET-MUP PEN-FOX-ERY-MUP PEN-FOX-GEN-TOB-CIP PEN-ERY-MUP	*mecA*, *tet*(K), *mupA* *blaZ*, *mecA*, *msr*(A), *mph*(C), *mupA* *blaZ*, *mecA*, *aac6′-aph2″* *blaZ*, *msr*(A), *mupA*
	72/27	*S. epidermidis* *S. hominis*	PEN-ERY Susceptible	*blaZ*, *msr*(A), *mph*(C) NT
	74/27	*S. epidermidis* *S. epidermidis* *S. lugdunensis*	PEN-SXT-CIP PEN-FOX-ERY-CIP PEN	*blaZ*, *dfrA*, *dfrG*, *blaZ*, *mecA*, *msr*(A) *blaZ*
Dog	4/2	*S. hominis* *S. epidermidis*	PEN-ERY-TOB ERY	*blaZ*, *msr*(A), *ant4′* *msr*(A)
	18/8	*S. haemolyticus* *S. warneri* *S. warneri* *S. epidermidis* *S. epidermidis*	TET ERY-SXT ERY-SXT-CHL PEN-FOX-ERY-SXT PEN-FOX	*tet*(K), *tet*(M) *msr*(A), *dfrA* *msr*(A), *dfrA*, *cat*_pC221_ *blaZ*, *mecA*, *msr*(A), *dfrA* *blaZ*, *mecA*
	28/11	*S. simulans* *S. epidermidis* *S. pasteuri*	Susceptible PEN Susceptible	NT *blaZ* NT
	29/11	*S. cohnii* *S. simulans*	PEN-FOX-ERY-CLI^ind^ PEN	*blaZ*, *mecA*, *erm*(A) *blaZ*
	32/13	*S. haemolyticus* *S. epidermidis* *S. epidermidis* *S. epidermidis* *S. haemolyticus*	GEN-TOB FOX-ERY-CLI^ind^-MUP Susceptible FOX-ERY-MUP FOX-ERY-CLI^ind^-SXT-MUP	*aac6′-aph2″* *mecA*, *erm*(A), *mupA* NT *mecA*, *msr(A)*, *mupA* *mecA*, *erm*(A), *dfrA*, *mupA*
	59/21	*S. hominis* *S. warneri* *S. wareneri*	PEN PEN-ERY PEN	*blaZ* *blaZ*, *msr*(A) *blaZ*

CHL: Chloramphenicol; CLI: clindamycin; CLI^ind^: clindamycin inducible; CIP: ciprofloxacin; ERY: erythromycin; FOX: cefoxitin; GEN: gentamicin; LZD: linezolid; MUP: mupirocin; PEN: penicillin; SXT: sulfamethoxazole/trimethoprim; TET: tetracycline, TOB: tobramycin. NT: Not tested.

**Table 5:** Intra-household carriers of coagulase-negative staphylococci with similar AMR pattern and sequence type.

Household Code	Host ID Code	Species	AMR Phenotype	AMR Gene Detected	Sequence Type
3	Human 5	*S. epidermidis*	Susceptible	NT	ST59
	Dog 6	*S. epidermidis*	Susceptible	NT	ST59
6	Human 13	*S. epidermidis*	Susceptible	NT	ST166
	Dog 12	*S. epidermidis*	Susceptible	NT	ST166
11	Human 26	*S. epidermidis*	PEN	*blaZ*	ST5
	Dog 28	*S. epidermidis*	PEN	*blaZ*	ST88
13	Dog 32	*S. epidermidis*	Susceptible	NT	ST61
	Human 33	*S. epidermidis*	Susceptible	NT	ST61
	Human 34	*S. epidermidis*	Susceptible	NT	ST73
19	Human 50	*S. epidermidis*	Susceptible	NT	ST85
	Hunan 51	*S. epidermidis*	CLI	*vga*(A)	ST278
	Dog 53	*S. epidermidis*	CLI	*vga*(A)	ST278

CLI: clindamycin; PEN: penicillin; NT: not tested.

## Data Availability

All the data derived from this study were comprehensively presented in this article. However, additional information may be requested from the corresponding author (C.T.).

## Supplementary Materials

The following supporting information can be downloaded at: https://www.mdpi.com/article/10.3390/pathogens13030229/s1, Table S1: Genes and primer sequences utilized for all PCRs in this study. References [40,41,42,43,44,45,46,47,48,49,50,51,52,53,54,55,56,57] are only cited in the Supplementary Materials. Figure S1a: Amino acid substitutions on 50S ribo-somal protein L3 of the linezolid-resistant *S. epidermidis* ST35. Figure S1b: Amino acid substitution on 50S ribosomal protein L4 of the linezolid-resistant *S. epidermidis* ST35.

## References

[1] França A., Gaio V., Lopes N., Melo L.D.R. (2021). Virulence Factors in Coagulase-Negative Staphylococci. Pathogens.

[2] Argemi X., Hansmann Y., Prola K., Prévost G. (2019). Coagulase-Negative Staphylococci Pathogenomics. Int. J. Mol. Sci..

[3] Severn M.M., Horswill A.R. (2023). *Staphylococcus epidermidis* and its dual lifestyle in skin health and infection. Nat. Rev. Microbiol..

[4] Joubert I.A., Otto M., Strunk T., Currie A.J. (2022). Look Who’s Talking: Host and Pathogen Drivers of *Staphylococcus epidermidis* Virulence in Neonatal Sepsis. Int. J. Mol. Sci..

[5] Widerström M., Stegger M., Johansson A., Gurram B.K., Larsen A.R., Wallinder L., Edebro H., Monsen T. (2022). Heterogeneity of *Staphylococcus epidermidis* in prosthetic joint infections: Time to reevaluate microbiological criteria?. Eur. J. Clin. Microbiol. Infect. Dis..

[6] Heilbronner S., Foster T.J. (2020). *Staphylococcus lugdunensis*: A Skin Commensal with Invasive Pathogenic Potential. Clin. Microb. Rev..

[7] Aldman M.H., Rasmussen M., Olaison L., Påhlman L.I. (2021). Endocarditis due to *Staphylococcus lugdunensis*-a retrospective national registry-based study. Eur. J. Clin. Microbiol. Infect. Dis..

[8] Heilbronner S. (2021). *Staphylococcus* *lugdunensis*. Trends Microbiol..

[9] Fernández-Fernández R., Lozano C., Ruiz-Ripa L., Robredo B., Azcona-Gutiérrez J.M., Alonso C.A., Aspiroz C., Zarazaga M., Torres C. (2022). Antimicrobial Resistance and Antimicrobial Activity of *Staphylococcus lugdunensis* Obtained from Two Spanish Hospitals. Microorganisms.

[10] Kizerwetter-Świda M., Chrobak-Chmiel D., Rzewuska M. (2019). High-level mupirocin resistance in methicillin-resistant staphylococci isolated from dogs and cats. BMC Vet. Res..

[11] LoPinto A.J., Mohammed H.O., Ledbetter E.C. (2015). Prevalence and risk factors for isolation of methicillin-resistant *Staphylococcus* in dogs with keratitis. Vet. Ophthalmol..

[12] Bean D.C., Wigmore S.M., Wareham D.W. (2017). Draft Genome Sequence of a Canine Isolate of Methicillin-Resistant *Staphylococcus haemolyticus*. Genome Announc..

[13] Marincola G., Liong O., Schoen C., Abouelfetouh A., Hamdy A., Wencker F.D.R., Marciniak T., Becker K., Köck R., Ziebuhr W. (2021). Antimicrobial resistance profiles of coagulase-Negative Staphylococci in community-based healthy individuals in Germany. Front. Public Health.

[14] Bowler P., Murphy C., Wolcott R. (2020). Biofilm exacerbates antibiotic resistance: Is this a current oversight in antimicrobial stewardship?. Antimicrob. Resis Infect. Contr..

[15] Grazul M., Balcerczak E., Sienkiewicz M. (2023). Analysis of the Presence of the Virulence and Regulation Genes from *Staphylococcus aureus* (*S. aureus*) in Coagulase Negative Staphylococci and the Influence of the Staphylococcal Cross-Talk on Their Functions. Int. J. Environ. Res. Public Health.

[16] Gostev V., Leyn S., Kruglov A., Likholetova D., Kalinogorskaya O., Baykina M., Dmitrieva N., Grigorievskaya Z., Priputnevich T., Lyubasovskaya L. (2021). Global Expansion of Linezolid-Resistant Coagulase-Negative Staphylococci. Front. Microbiol..

[17] Heilmann C., Ziebuhr W., Becker K. (2019). Are coagulase-negative staphylococci virulent?. Clin. Microbiol. Infect..

[18] Abdullahi I.N., Lozano C., Zarazaga M., Saidenberg A.B.S., Stegger M., Torres C. (2023). Clonal relatedness of coagulase-positive staphylococci among healthy dogs and dog-owners in Spain. Detection of multidrug-resistant-MSSA-CC398 and novel linezolid-resistant-MRSA-CC5. Front. Microbiol..

[19] Gómez-Sanz E., Ceballos S., Ruiz-Ripa L., Zarazaga M., Torres C. (2019). Clonally Diverse Methicillin and Multidrug Resistant Coagulase Negative Staphylococci Are Ubiquitous and Pose Transfer Ability between Pets and Their Owners. Front. Microbiol..

[20] Torres-Sangiao E., Leal Rodriguez C., García-Riestra C. (2021). Application and Perspectives of MALDI-TOF Mass Spectrometry in Clinical Microbiology Laboratories. Microorganisms.

[21] Breakpoint Tables for Interpretation of MICs and Zone Diameters Version 12.0. Sweden: European Committee on Antimicrobial Susceptibility Testing. 2022. European Committee on Antimicrobial Susceptibility Testing. EUCAST Clinical Breakpoint Tables for Interpretation of MICs and Zone Diameters, Version 12.0. https://www.eucast.org/fileadmin/src/media/PDFs/EUCAST_files/Breakpoint_tables/v_14.0_Breakpoint_Tables.pdf.

[22] Magiorakos A.P., Srinivasan A., Carey R.B., Carmeli Y., Falagas M.E., Giske C.G., Harbarth S., Hindler J.F., Kahlmeter G., Olsson-Liljequist B. (2012). Multidrug-resistant, extensively drug-resistant and pandrug-resistant bacteria: An international expert proposal for interim standard definitions for acquired resistance. Clin. Microbiol. Infect..

[23] Feßler A.T., Wang Y., Burbick C.R. (2023). Antimicrobial susceptibility testing in veterinary medicine: Performance, interpretation of results, best practices and pitfalls. One Health Adv..

[24] Zhang K., McClure J.A., Elsayed S., Louie T., Conly J.M. (2005). Novel multiplex PCR assay for characterization and concomitant subtyping of staphylococcal cassette chromosome *mec* types I to V in methicillin-resistant *Staphylococcus aureus*. J. Clin. Microbiol..

[25] Suepaul S., Georges K., Unakal C., Boyen F., Sookhoo J., Ashraph K., Yusuf A., Butaye P. (2021). Determination of the frequency, species distribution and antimicrobial resistance of staphylococci isolated from dogs and their owners in Trinidad. PLoS ONE.

[26] Burke T.L., Rupp M.E., Fey P.D. (2023). *Staphylococcus* *epidermidis*. Trends Microbiol..

[27] Han J.I., Yang C.H., Park H.M. (2016). Prevalence and risk factors of *Staphylococcus* spp. carriage among dogs and their owners: A cross-sectional study. Vet. J..

[28] Štempelová L., Kubašová I., Bujňáková D., Kačírová J., Farbáková J., Maďar M., Karahutová L., Strompfová V. (2021). Distribution and Characterization of Staphylococci Isolated from Healthy Canine Skin. Top Compan. Animal. Med..

[29] Rook K.A., Brown D.C., Rankin S.C., Morris D.O. (2012). Case-control study of *Staphylococcus lugdunensis* infection isolates from small companion animals. Vet. Dermatol..

[30] Abadi M.I.M., Moniri R., Khorshidi A., Piroozmand A., Mousavi S.G.A., Dastehgoli K. (2015). Molecular characteristics of nasal carriage methicillin-resistant coagulase negative staphylococci in school students. Jundishapur J. Microbiol..

[31] Xu Z., Shah H.N., Misra R., Chen J., Zhang W., Liu Y. (2018). The prevalence, antibiotic resistance and *mecA* characterization of coagulase negative staphylococci recovered from non-healthcare settings in London, UK. Antimicrob. Resist. Infect. Control.

[32] Lebeaux D., Barbier F., Angebault C., Benmahdi L., Ruppé E., Felix B., Gaillard K., Djossou F., Epelboin L., Dupont C. (2012). Evolution of nasal carriage of methicillin-resistant coagulase-negative staphylococci in a remote population. Antimicrob. Agents Chemotherap..

[33] Jamaluddin T.Z., Kuwahara-Arai K., Hisata K., Terasawa M., Cui L., Baba T., Sotozono C., Kinoshita S., Ito T., Hiramatsu K. (2008). Extreme genetic diversity of methicillin-resistant *Staphylococcus epidermidis* strains disseminated among healthy Japanese children. J. Clin Microbiol..

[34] Morgenstern M., Erichsen C., Hackl S., Mily J., Militz M., Friederichs J., Hungerer S., Bühren V., Moriarty T.F., Post V. (2016). Antibiotic Resistance of Commensal *Staphylococcus aureus* and Coagulase-Negative Staphylococci in an International Cohort of Surgeons: A Prospective Point-Prevalence Study. PLoS ONE.

[35] Mahfouz A.A., Said H.S., Elfeky S.M., Shaaban M.I. (2023). Inhibition of erythromycin and erythromycin-Induced resistance among *Staphylococcus aureus* clinical isolates. Antibiotics.

[36] Conner J.G., Smith J., Erol E., Locke S., Phillips E., Carter C.N., Odoi A. (2018). Temporal trends and predictors of antimicrobial resistance among *Staphylococcus* spp. isolated from canine specimens submitted to a diagnostic laboratory. PLoS ONE.

[37] Allport J., Choudhury R., Bruce-Wootton P., Reed M., Tate D., Malviya A. (2022). Efficacy of mupirocin, neomycin and octenidine for nasal *Staphylococcus aureus* decolonisation: A retrospective cohort study. Antimicrob. Resist. Infect. Contr..

[38] Lord J., Millis N., Jones R.D., Johnson B., Kania S.A., Odoi A. (2022). Patterns of antimicrobial, multidrug and methicillin resistance among *Staphylococcus* spp. isolated from canine specimens submitted to a diagnostic laboratory in Tennessee, USA: A descriptive study. BMC Vet. Res..

[39] Coustillères F., Renault V., Corvec S., Dupieux C., Simões P.M., Lartigue M.F., Plouzeau-Jayle C., Tande D., Lamoureux C., Lemarié C. (2023). Clinical, Bacteriological, and Genetic Characterization of Bone and Joint Infections Involving Linezolid-Resistant *Staphylococcus epidermidis*: A Retrospective Multicenter Study in French Reference Centers. Microbiol. Spectr..

[40] Schnellmann C., Gerber V., Rossano A., Jaquier V., Panchaud Y., Doherr M.G., Thomann A., Straub R., Perreten V. (2006). Presence of new *mecA* and *mph(C*) variants conferring antibiotic resistance in *Staphylococcus* spp. isolated from the skin of horses before and after clinic admission. J. Clin. Microbiol..

[41] Poulsen A.B., Skov R., Pallesen L.V. (2003). Detection of methicillin resistance in coagulase-negative staphylococci and in staphylococci directly from simulated blood cultures using the EVIGENE MRSA Detection Kit. J. Antimicrob. Chemotherap..

[42] Sutcliffe J., Grebe T., Tait-Kamradt A., Wondrack L. (1996). Detection of erythromycin-resistant determinants by PCR. Antimicrob. Agent Chemotherap..

[43] Gómez-Sanz E., Torres C., Lozano C., Fernández-Pérez R., Aspiroz C., Ruiz-Larrea F., Zarazaga M. (2010). Detection, molecular characterization, and clonal diversity of methicillin-resistant *Staphylococcus aureus* CC398 and CC97 in Spanish slaughter pigs of different age groups. Foodborne Path. Dis..

[44] Wondrack L., Massa M., Yang B.V., Sutcliffe J. (1996). Clinical strain of *Staphylococcus aureus* inactivates and causes efflux of macrolides. Antimicrob. Agent Chemotherap..

[45] Lina G., Quaglia A., Reverdy M.E., Leclercq R., Vandenesch F., Etienne J. (1999). Distribution of genes encoding resistance to macrolides, lincosamides, and streptogramins among staphylococci. Antimicrob. Agent Chemotherap..

[46] Bozdogan B., Berrezouga L., Kuo M.S., Yurek D.A., Farley K.A., Stockman B.J., Leclercq R. (1999). A new resistance gene, *linB,* conferring resistance to lincosamides by nucleotidylation in *Enterococcus faecium* HM1025. Antimicrob. Agent Chemotherap..

[47] Lozano C., Aspiroz C., Rezusta A., Gómez-Sanz E., Simon C., Gómez P., Ortega C., Revillo M.J., Zarazaga M., Torres C. (2012). Identification of novel *vga(A)*-carrying plasmids and a Tn5406-like transposon in meticillin-resistant *Staphylococcus aureus* and *Staphylococcus epidermidis* of human and animal origin. Int. J. Antimicrob. Agent.

[48] Van de Klundert J., Vliegenthart J. (1993). PCR detection of genes coding for aminoglycoside-modifying enzymes. Diagnostic Molecular Microbiology: Principles and Applications.

[49] Aarestrup F.M., Agerso Y., Gerner-Smidt P., Madsen M., Jensen L.B. (2020). Comparison of antimicrobial resistance phenotypes and resistance genes in *Enterococcus faecalis* and *Enterococcus faecium* from humans in the community, broilers, and pigs in Denmark. Diagn. Mcrobiol. Infect Dis..

[50] Kehrenberg C., Schwarz S. (2005). Florfenicol-chloramphenicol exporter gene *fexA* is part of the novel transposon Tn558. Antimicrob. Agent Chemotherap..

[51] Liu H., Wang Y., Wu C., Schwarz S., Shen Z., Jeon B., Ding S., Zhang Q., Shen J. (2012). A novel phenicol exporter gene, *fexB,* found in enterococci of animal origin. J. Antimicrob. Chemotherap..

[52] Kehrenberg C., Schwarz S. (2006). Distribution of florfenicol resistance genes *fexA* and *cfr* among chloramphenicol-resistant Staphylococcus isolates. Antimicrob. Agent Chemotherap..

[53] Lee S.M., Huh H.J., Song D.J., Shim H.J., Park K.S., Kang C.I., Ki C.S., Lee N.Y. (2017). Resistance mechanisms of linezolid-nonsusceptible enterococci in Korea: Low rate of 23S rRNA mutations in *Enterococcus faecium*. J. Med. Microbiol..

[54] Ruiz-Ripa L., Feßler A.T., Hanke D., Eichhorn I., Azcona-Gutiérrez J.M., Pérez-Moreno M.O., Seral C., Aspiroz C., Alonso C.A., Torres L. (2020). Mechanisms of Linezolid Resistance Among Enterococci of Clinical Origin in Spain-Detection of *optrA*- and *cfr*(D)-Carrying *E. faecalis*. Microorganisms.

[55] Wang Y., Lv Y., Cai J., Schwarz S., Cui L., Hu Z., Zhang R., Li J., Zhao Q., He T. (2015). A novel gene, *optrA,* that confers transferable resistance to oxazolidinones and phenicols and its presence in *Enterococcus faecalis* and *Enterococcus faecium* of human and animal origin. J. Antimicrobial. Chemotherap..

[56] Udo E.E., Al-Sweih N., Noronha B.C. (2003). A chromosomal location of the mupA gene in Staphylococcus aureus expressing high-level mupirocin resistance. J. Antimicrob. Chemotherap..

[57] Thomas J.C., Vargas M.R., Miragaia M., Peacock S.J., Archer G.L., Enright M.C. (2007). Improved multilocus sequence typing scheme for *Staphylococcus epidermidis*. J. Clin. Miicrobiol..

