# Supplementary material for: Genetic Diversification and Resistome of Coagulase-Negative Staphylococci from Nostrils of Healthy Dogs and Dog-Owners in La Rioja, Spain
Source: Pathogens. 2024 Mar 5;13(3):229. doi: 10.3390/pathogens13030229 (PMC10974962; doi:10.3390/pathogens13030229)
Supplement: Supplementary file 1 [file pathogens-13-00229-s001.zip › ad-Pathogens pets CoNS-Supplementary Table S1-FINAL.pdf]

Table S1. Genes and primers' sequences utilized for all PCRs in this study.

Gene	Primers' oligonucleotide (5'⇒3')	Amplicon size	Reference
**Antimicrobial resistance (AMR) genes**			
blaZ	F: CAGTTCACATGCCAAAGAG	772 bp	[1]
	R: TACACTCTTGGCGGTTTC		
mecA	F: GGGATCATAGCGTCATTATTC	527 bp	[2]
	R: AACGATTGTGACACGATAGCC		
ermA	F: TCTAAAAAGCATGTAAAAGAA	645 bp	[3]
	R: CTTCGATAGTTTATTAATATTAG		
ermB	F: GAAAAGTACTCAACCAAATA	639 bp	[3]
	R: AGTAACGGTACTTAAATTGTTTA		
ermC	F: TCAAAACATAATATAGATAAA	642 bp	[3]
	R: GCTAATATTGTTTAAATCGTCAAT		
ermT	F: CCGCCATTGAAATAGATCCT	200 bp	[4]
	R: TTCTGTAGCTGTGCTTTCAAAAA		
mphC	F: ATGACTCGACATAATGAAAT	900 bp	[1]
	R: CTACTCTTTCATACCTAACTC		
msrA	F: GCAAATGGTGTAGGTAAGACAACT	399 bp	[5]
	R: ATCATGTGATGTAAACAAAAT		
lnuA	F: GGTGGCTGGGGGGTAGATGTATTAACTGG	323 bp	[6]
	R: GCTTCTTTTGAAATACATGGTATTTTTCGATC		
lnuB	F: CCTACCTATTGTTTGTGGAA	499 bp	[7]
	R: ATAACGTTACTCTCCTATTC		
vgaA	F: AGTGGTGGTGAAGTAACACG	1264 pb	[8]
	R: GGTTCAATACTCAATCGACTGAG		
aac6'-aph2''	F: CCAAGAGCAATAAGGGCATA	220 bp	[9]
	R: CACTATCATAACCACTACCG		
ant4'	F: GCAAGGACCGACAACATTTC	165 bp	[9]
	R: TGGCACAGATGGTCATAACC		

tet(L)	F: CATTTGGTCTTATTGGATCG<br>R: ATTACACTTCCGATTTCGG	456 bp	[10]
tet(M)	F: GTTAAATAGTGTTCTTGGAG<br>R: CTAAGATATGGCTCTAACAA	576 bp	[10]
tet(K)	F: TTAGGTGAAGGGTTAGGTCC<br>R: GCAAACTCATTCCAGAAGCA	697 bp	[10]
dfrA	F: CCTTGGCACTTACCAAATG<br>R: CTGAAGATTCGACTTCCC	374 bp	[1]
dfrD	F: TTCTTTAATTGTTGCGATGG<br>R: TTAACGAATTCTCTCATATATATG	582 bp	[1]
dfrG	F: TCGGAAGAGCCTTACCTGACAGAA<br>R: CCCTTTTTGGGCAAATACCTCATTCCA	323 bp	[4]
dfrK	F: GAGAATCCCAGAGGATTGGG<br>R: CAAGAAGCTTTTCGCTCATAAA	423 bp	[4]
cat_{pC221}	F: ATTTATGCAATTATGGAAGTTG<br>R: TGAAGCATGGTAACCATCAC	434 bp	[1]
cat_{pC223}	F: GAATCAAATGCTAGTTTTAACTC<br>R: ACATGGTAACCATCACATAC	283 bp	[1]
cat_{pC194}	F: CGACTTTTAGTATAACCACAGA<br>R: GCCAGTCATTAGGCCTAT	570 bp	[1]
catA	F: GGATATGAAATTTATCCCTC<br>R: CAATCATCTACCCTATGAAT	505 bp	[10]
fexA	F: GTACTTGTAGGTGCAATTACGGCTGA<br>R: CGCATCTGAGTAGGACATAGCGTC	1272 bp	[11]
fexB	F: TTCCCACTATTGGTGAAAGGAT<br>R: GCAATTCCCTTTTATGGACGTT	816 bp	[12]
cfr	F: TGAAGTATAAAGCAGGTTGGGAGTCA<br>R: ACCATATAATTGACCACAAGCAGC	746 bp	[13]
cfrB	F: TGAGCATATACGAGTAACCTCAAGA<br>R: CGCAAGCAGCGTCTATATCA	293 bp	[14]
cfrD	F: AGAAGTCGCAACAAGTGAGGA<br>R: GCAACTGCATGAGTCAAAGAA	595 bp	[15]
optrA	F: AGGTGGTCAGCGAACTAA	1395 bp	[16]

	R: ATCAACTGTTCCCATTCA		
poxtA	F: TCAATGCAGAGCAGGAAGCA	791 bp	[15]
	R: GGTGGATTTACCGACACCGT		
mupA	F: CCCATGGCTTACCAGTTGA	419 pb	[17]
	R: CCATGGAGCACTATCCGAA		
***S. epidermidis* housekeeping alleles for MLST**			
arcC	F: TGTGATGAGCACGCTACCGTTAG	465 pb	[18]
	R: TCCAAGTAAACCCATCGGTCTG		
aroE	F: CATTGGATTACCTCTTTGTTCAGC	430 pb	[18]
	R: CAAGCGAAATCTGTTGGGG		
gti	F: CAGCCAATTCTTTTATGACTTTT	438 pb	[18]
	R: GTGATTAAAGGTATTGATTTGAAT		
mutS	F: GATATAAGAATAAGGGTTGTGAA	412 bp	[18]
	R: GTAATCGTCTCAGTTATCATGTT		
pyrR	F: GTTACTAATACTTTTGCTGTGTTT	428 bp	[18]
	R: GTAGAATGTAAAGAGACTAAAATGAA		
tpi	F: ATCCAATTAGACGCTTTAGTAAC	424 bp	[18]
	R: TTAATGATGCGCCACCTACA		
yqiL	F: CACGCATAGTATTAGCTGAAG	416 bp	[18]
	R: CTAATGCCTTCATCTTGAGAAATAA		

1. Schnellmann, C.; Gerber, V.; Rossano, A.; Jaquier, V.; Panchaud, Y.; Doherr, M. G.; Thomann, A.; Straub, R.; & Perreten, V. Presence of new *mecA* and *mph(C)* variants conferring antibiotic resistance in *Staphylococcus* spp. isolated from the skin of horses before and after clinic admission. *J Clin Microbiol*, **2006**; *44*(12), 4444–4454. https://doi.org/10.1128/JCM.00868-06
2. Poulsen, A. B.; Skov, R.; & Pallesen, L. V. Detection of methicillin resistance in coagulase-negative staphylococci and in staphylococci directly from simulated blood cultures using the EVIGENE MRSA Detection Kit. *J Antimicrob Chemotherap*, **2003;** *51*(2), 419–421. https://doi.org/10.1093/jac/dkg084

3. Sutcliffe, J.; Grebe, T.; Tait-Kamradt, A.; & Wondrack, L. Detection of erythromycin-resistant determinants by PCR. *Antimicrob agent Chemotherap*, **1996**; *40*(11), 2562–2566. https://doi.org/10.1128/AAC.40.11.2562
4. Gómez-Sanz, E.; Torres, C.; Lozano, C.; Fernández-Pérez, R.; Aspiroz, C.; Ruiz-Larrea, F.; & Zarazaga, M. Detection, molecular characterization, and clonal diversity of methicillin-resistant *Staphylococcus aureus* CC398 and CC97 in Spanish slaughter pigs of different age groups. *Foodborne Path Dis*, **2010**; *7*(10), 1269–1277. https://doi.org/10.1089/fpd.2010.0610
5. Wondrack, L.; Massa, M.; Yang, B. V.; & Sutcliffe, J. Clinical strain of *Staphylococcus aureus* inactivates and causes efflux of macrolides. *Antimicrob Agent Chemotherap*, 1**996**; *40*(4), 992–998. https://doi.org/10.1128/AAC.40.4.992
6. Lina, G.; Quaglia, A.; Reverdy, M. E.; Leclercq, R.; Vandenesch, F.; & Etienne, J. Distribution of genes encoding resistance to macrolides, lincosamides, and streptogramins among staphylococci. *Antimicrob Agent Chemotherap*, **1999;** *43*(5), 1062–1066. https://doi.org/10.1128/AAC.43.5.1062
7. Bozdogan, B.; Berrezouga, L.; Kuo, M. S.; Yurek, D. A.; Farley, K. A.; Stockman, B. J.; & Leclercq, R. A new resistance gene, *linB,* conferring resistance to lincosamides by nucleotidylation in *Enterococcus faecium* HM1025. *Antimicrob Agent Chemotherap,* **1999;** *43*(4), 925–929. https://doi.org/10.1128/AAC.43.4.925
8. Lozano, C.; Aspiroz, C.; Rezusta, A.; Gómez-Sanz, E.; Simon, C.; Gómez, P.; Ortega, C.; Revillo, M. J.; Zarazaga, M.; & Torres, C. Identification of novel *vga(A)*-carrying plasmids and a Tn5406-like transposon in meticillin-resistant *Staphylococcus aureus* and *Staphylococcus epidermidis* of human and animal origin. *Int J Antimicrob Agent*, **2012;** *40*(4), 306–312. https://doi.org/10.1016/j.ijantimicag.2012.06.009
9. Van de Klundert, J.; Vliegenthart, J. PCR detection of genes coding for aminoglycoside-modifying enzymes, in: Diagnostic Molecular Microbiology. Principles and Applications. **1993**; 547– 552. https://doi.org/10.1023/A:1016601629518
10. Aarestrup, F. M.; Agerso, Y.; Gerner-Smidt, P.; Madsen, M.; & Jensen, L. B. Comparison of antimicrobial resistance phenotypes and resistance genes in *Enterococcus faecalis* and *Enterococcus faecium* from humans in the community, broilers, and pigs in Denmark. *Diagn Mcrobiol Infect Dis*, **2020**; *37*(2), 127–137. https://doi.org/10.1016/s0732-8893(00)00130-9

11. Kehrenberg, C.; & Schwarz, S. Florfenicol-chloramphenicol exporter gene *fexA* is part of the novel transposon Tn558. *Antimicrob Agent Chemotherap*, **2005**; *49*(2), 813–815. https://doi.org/10.1128/AAC.49.2.813-815.2005
12. Liu, H.; Wang, Y.; Wu, C.; Schwarz, S.; Shen, Z.; Jeon, B.; Ding, S.; Zhang, Q.; & Shen, J. A novel phenicol exporter gene, *fexB*, found in enterococci of animal origin. *J Antimicrob Chemotherap*, **2012;** *67*(2), 322–325. https://doi.org/10.1093/jac/dkr481
13. Kehrenberg, C.; & Schwarz, S. Distribution of florfenicol resistance genes *fexA* and *cfr* among chloramphenicol-resistant Staphylococcus isolates. *Antimicrob Agent Chemotherap*, **2006;** *50*(4), 1156–1163. https://doi.org/10.1128/AAC.50.4.1156-1163.2006
14. Lee, S. M.; Huh, H. J.; Song, D. J.; Shim, H. J.; Park, K. S.; Kang, C. I.; Ki, C. S.; & Lee, N. Y. Resistance mechanisms of linezolid-nonsusceptible enterococci in Korea: low rate of 23S rRNA mutations in *Enterococcus faecium*. *J Med Microbiol*, **2017**;*66*(12), 1730–1735. https://doi.org/10.1099/jmm.0.000637
15. Ruiz-Ripa, L.; Feßler, A. T.; Hanke, D.; Eichhorn, I.; Azcona-Gutiérrez, J. M.; Pérez-Moreno, M. O.; Seral, C.; Aspiroz, C.; Alonso, C. A.; Torres, L.; Alós, J. I.; Schwarz, S.; & Torres, C. Mechanisms of Linezolid Resistance Among Enterococci of Clinical Origin in Spain-Detection of *optrA*- and *cfr*(D)-Carrying *E.faecalis*. *Microorganisms*, **2020**; *8*(8), 1155. https://doi.org/10.3390/microorganisms8081155
16. Wang, Y.; Lv, Y.; Cai, J.; Schwarz, S.; Cui, L.; Hu, Z.; Zhang, R.; Li, J.; Zhao, Q.; He, T.; Wang, D.; Wang, Z.; Shen, Y.; Li, Y.; Feßler, A. T.; Wu, C.; Yu, H.; Deng, X.; Xia, X.; & Shen, J. A novel gene, *optrA*, that confers transferable resistance to oxazolidinones and phenicols and its presence in *Enterococcus faecalis* and *Enterococcus faecium* of human and animal origin. *J Antimicrobial Chemotherap*, **2015**; *70*(8), 2182–2190. https://doi.org/10.1093/jac/dkv116
17. Udo, E. E.; Al-Sweih, N.; & Noronha, B. C. (2003). A chromosomal location of the mupA gene in Staphylococcus aureus expressing high-level mupirocin resistance. *J Antimicrob Chemotherap*, **2003**; *51*(5), 1283–1286. https://doi.org/10.1093/jac/dkg188
18. Thomas, J. C.; Vargas, M. R.; Miragaia, M.; Peacock, S. J.; Archer, G. L.; & Enright, M. C. Improved multilocus sequence typing scheme for *Staphylococcus epidermidis*. *J Clin Miicrobiol*, **2007**; *45*(2), 616–619. https://doi.org/10.1128/JCM.01934-06
