# Supplementary figures and images for: Genetic Diversification and Resistome of Coagulase-Negative Staphylococci from Nostrils of Healthy Dogs and Dog-Owners in La Rioja, Spain
Source: Pathogens. 2024 Mar 5;13(3):229. doi: 10.3390/pathogens13030229 (PMC10974962; doi:10.3390/pathogens13030229)

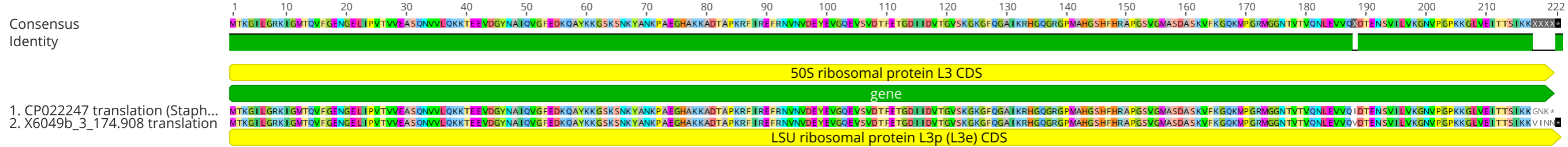

Supplement: Supplementary file 1 [file pathogens-13-00229-s001.zip › ab-Pathogens-pets CoNS-Supplementary Figure S1a-FINAL.pdf]

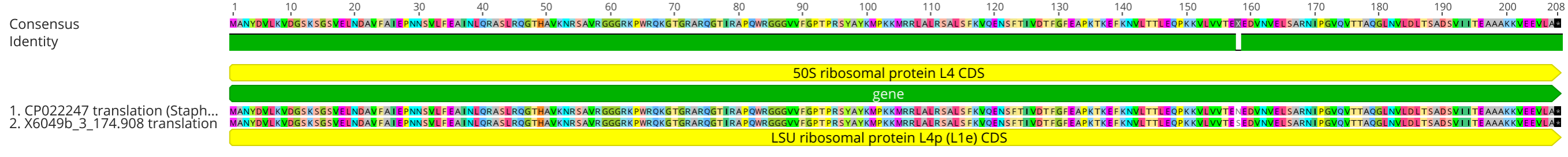

Supplement: Supplementary file 1 [file pathogens-13-00229-s001.zip › ac-Pathogens pets CoNS-Supplementary Figure S1b-FINAL.pdf]
